# Validation of amyloid PET positivity thresholds in centiloids: a multisite PET study approach

**DOI:** 10.1186/s13195-021-00836-1

**Published:** 2021-05-10

**Authors:** Sarah K. Royse, Davneet S. Minhas, Brian J. Lopresti, Alice Murphy, Tyler Ward, Robert A. Koeppe, Santiago Bullich, Susan DeSanti, William J. Jagust, Susan M. Landau, for the Alzheimer’s Disease Neuroimaging Initiative

**Affiliations:** 1 Department of Radiology, University of Pittsburgh, Pittsburgh, PA USA; 2 Helen Wills Neuroscience Institute, University of California Berkeley, Berkeley, CA USA; 3 Division of Nuclear Medicine, University of Michigan, Ann Arbor, MI USA; 4 Life Molecular Imaging GmbH, Berlin, Germany; 5 Life Molecular Imaging Inc, Boston, MA USA

**Keywords:** Centiloid, Florbetaben, Florbetapir, Amyloid imaging, Beta-amyloid, Standardization, Alzheimer’s disease

## Abstract

**Background:**

Inconsistent positivity thresholds, image analysis pipelines, and quantitative outcomes are key challenges of multisite studies using more than one β-amyloid (Aβ) radiotracer in positron emission tomography (PET). Variability related to these factors contributes to disagreement and lack of replicability in research and clinical trials. To address these problems and promote Aβ PET harmonization, we used [^18^F]florbetaben (FBB) and [^18^F]florbetapir (FBP) data from the Alzheimer’s Disease Neuroimaging Initiative (ADNI) to derive (1) standardized Centiloid (CL) transformations and (2) internally consistent positivity thresholds based on separate young control samples.

**Methods:**

We analyzed Aβ PET data using a native-space, automated image processing pipeline that is used for PET quantification in many large, multisite AD studies and trials and made available to the research community. With this pipeline, we derived SUVR-to-CL transformations using the Global Alzheimer’s Association Interactive Network data; we used reference regions for cross-sectional (whole cerebellum) and longitudinal (subcortical white matter, brain stem, whole cerebellum) analyses. Finally, we developed a FBB positivity threshold using an independent young control sample (*N*=62) with methods parallel to our existing FBP positivity threshold and validated the FBB threshold using a data-driven approach in ADNI participants (*N*=295).

**Results:**

The FBB threshold based on the young sample (1.08; 18 CL) was consistent with that of the data-driven approach (1.10; 21 CL), and the existing FBP threshold converted to CL with the derived transformation (1.11; 20 CL). The following equations can be used to convert whole cerebellum- (cross-sectional) and composite- (longitudinal) normalized FBB and FBP data quantified with the native-space pipeline to CL units:

[^18^F]FBB: CL_whole cerebellum_ = 157.15 × SUVR_FBB_ − 151.87; threshold=1.08, 18 CL

[^18^F]FBP: CL_whole cerebellum_ = 188.22 × SUVR_FBP_ − 189.16; threshold=1.11, 20 CL

[^18^F]FBB: CL_composite_ = 244.20 × SUVR_FBB_ − 170.80

[^18^F]FBP: CL_composite_ = 300.66 × SUVR_FBP_ − 208.84

**Conclusions:**

FBB and FBP positivity thresholds derived from independent young control samples and quantified using an automated, native-space approach result in similar CL values. These findings are applicable to thousands of available and anticipated outcomes analyzed using this pipeline and shared with the scientific community. This work demonstrates the feasibility of harmonized PET acquisition and analysis in multisite PET studies and internal consistency of positivity thresholds in standardized units.

**Supplementary Information:**

The online version contains supplementary material available at 10.1186/s13195-021-00836-1.

## Background

Positron emission tomography (PET) imaging has played an integral role in Alzheimer’s disease (AD) neuroimaging research by yielding precise in vivo measurement of β-amyloid (Aβ) pathology [1]. Still, Aβ PET studies are limited by the variability that can be introduced through non-specific binding properties of radiotracers [2], scanner and reconstruction differences, and variations in analysis pipelines [3, 4]. This variability ultimately frustrates efforts to combine data for meta-analyses and multicenter studies [5], track longitudinal changes in Aβ burden [6], and establish universal cut points for Aβ positivity [5]. In light of these issues, Klunk et al. [5] developed the Centiloid (CL) method, which standardizes total Aβ burden assessed with PET imaging agents by (1) establishing a standard analysis pipeline for quantifying cortical standardized uptake value ratios (SUVRs) and (2) converting SUVRs across various Aβ radiotracers and analysis methods to a common scale. In other words, investigators can choose to express their data in CL units either by using the standard CL analysis pipeline or by cross-calibrating their data against previously validated data, ultimately yielding linear equations for transforming their data into CL units.

The Alzheimer’s Disease Neuroimaging Initiative (ADNI) is an ongoing, multisite observational study of AD. Presently, thousands of [^18^F]florbetaben (FBB; 90–110 min) and [^18^F]florbetapir (FBP; 50–60 min) SUVRs are available to the scientific community through ADNI’s database website (http://adni.loni.usc.edu), with more anticipated to be collected and shared under the current protocol (ADNI-3). Furthermore, ADNI-compatible PET acquisition and processing methods are being implemented in other ongoing multisite AD studies such as the Longitudinal Early-Onset Alzheimer's Disease Study (LEADS) [7] and the Standardized Centralized Alzheimer’s and Related Dementias Neuroimaging (SCAN) project [8]. Data from ADNI and other studies with compatible protocols are also frequently used by the scientific community. As of 2020, ADNI data have been downloaded more than 100 million times by users across workforce sectors [9], with ADNI additionally having been credited in over 1800 scholarly publications. Taken together, and in conjunction with the Aβ PET issues described above, a standard outcome measurement like the CL method holds the potential to benefit a large number of PET studies; this would enable harmonization of Aβ PET across various sites, scanners, and tracers, ultimately increasing statistical power for research studies and clinical trials that wish to use Aβ PET imaging for prediction or outcome measures.

To promote such harmonization, the primary objectives of this work were to (1) create direct CL conversion equations for FBB and FBP SUVRs derived from ADNI processing methods to facilitate both cross-sectional and longitudinal analyses and (2) establish positivity thresholds for ADNI-derived FBB and FBP SUVRs based on independent young control samples. We accomplished our first objective by analyzing FBB and FBP datasets available on the Global Alzheimer’s Association Interactive Network (GAAIN) website (www.gain.org/centiloid-project) through ADNI’s automatic, native-space pipeline and subsequently following procedures necessary for level-2 analysis as described by Klunk et al. [5]. While linear equations for converting FBB and FBP SUVRs derived from the standard CL pipeline to CL units have been published [10, 11], these transformations are only valid when applied to data analyzed with the standard CL analysis pipeline; PET processing pipelines that use different analysis approaches, such as that of ADNI, change the quantification parameters and therefore the relationship of analysis outcomes to CL units. We defined CL conversions for ADNI-derived FBB and FBP SUVRs normalized to the whole cerebellum, for use in cross-sectional analyses, and normalized to a composite reference region (made up of eroded subcortical white matter, brainstem, and whole cerebellum), which has shown greater reliability for longitudinal analyses [12–15].

To accomplish our other primary objective, we used the CL conversion equations to calculate thresholds in CL units for FBB and FBP that are compatible with ADNI acquisition processing methods. For FBB, existing thresholds have been reported based on the detection of Aβ [16] or the separation of Aβ-positive patients and Aβ-negative controls [17, 18] but these thresholds were defined using pipelines that differ from that of ADNI, so they are not directly applicable to our data. In addition, these thresholds may be less sensitive to early increases in Aβ burden than techniques that emphasize the detection of Aβ relative to young individuals with no evidence of any Aβ. Thus, in order to define a FBB threshold that is congruent with the use of a young control sample as a standard of comparison, we examined FBB uptake in young healthy controls and validated this finding with a data-driven approach in ADNI participants using the updated ADNI PET pipeline. For FBP, we used a threshold (1.11) that was based on the upper 95% confidence interval of mean cortical FBP SUVRs relative to the whole cerebellum in a young control group [19] and transformed to the ADNI FreeSurfer (FS) pipeline initially using FS v5.3 [3]. Here, we validate this FBP threshold with the updated FS v7.1-based pipeline.

## Methods

### Subject cohorts

We examined FBB and FBP data from several cohorts to derive CL conversion equations and the positivity thresholds for each tracer.

#### Centiloid derivation cohort

Paired FBB (90–110 min) and ^11^C-Pittsburgh Compound-B (PiB) (50–70 min) PET scans with accompanying magnetic resonance images (MRIs) were obtained in 35 subjects (25 elderly, 10 young control) at Austin Hospital, Melbourne, Australia [10]. Concurrent FBB and PiB PET scans were acquired on either a Phillips Allegro PET camera or a Phillips TF64 PET/computed tomography (CT) scanner. Images acquired on the Allegro were processed with rotating Cs-137 point source attenuation correction and reconstructed using a 3D row-action maximum likelihood algorithm (RAMLA). Those that were collected on the TF64 used a CT for attenuation correction and a line-response RAMLA for reconstruction. All MRIs were acquired on a Siemens 3-T Trio using a T1 magnetization-prepared rapid acquisition gradient-echo (MPRAGE) sequence at a resolution of 1 × 1 × 1.2 mm voxels.

Paired FBP (50–60 min) and PiB (50–70 min) PET scans with corresponding MRIs were independently acquired in 46 subjects (33 elderly, 13 young control) by Avid Radiopharmaceuticals [11]. Corresponding FBP and PiB PET data were acquired on one of three scanners: a Siemens HR+ (collected in 2D mode and processed using a Ge-68 rod source and 2D-OSEM reconstruction), a Philips Gemini TF 64 (acquired in 3D mode and processed using CT for attenuation correction and a line-response RAMLA for reconstruction), or a GE Advance (collected in 2D mode and processed using a Ge-68 rod source and FORE-iterative reconstruction algorithm). All T1 MRIs were acquired on a 3T scanner.

We downloaded both PET and MRI datasets used in these analyses from the Global Alzheimer’s Association Interactive Network (GAAIN) website: http://www.gaain.org/centiloid-project.

#### FBB threshold derivation cohort

We used 62 young, cognitively normal control FBB scans and contemporaneous MRI scans acquired by Life Molecular Imaging (LMI; formerly Piramal Imaging) (age range 21–40; mean age 27.5 ± 5.1 years; 4 × 5 min frames, 100–120 min [20]). Hoffman phantom data were used to determine the smoothing required to achieve the same effective resolution as ADNI images (8mm^3^ FWHM). Images were further processed with the updated ADNI pipeline (see the “ADNI pipeline” section) to derive a FBB positivity threshold.

#### Threshold validation cohort

In the preparation of this article, we obtained data from the ADNI database (adni.loni.usc.edu). The ADNI was launched in 2003 as a public-private partnership, led by Principal Investigator Michael W. Weiner, MD. The primary goal of ADNI has been to test whether serial MRI, PET, other biological markers, and clinical and neuropsychological assessment can be combined to measure the progression of MCI and early AD.

We used 1292 ADNI-2 and ADNI-3 participants (487 cognitively normal, 585 mild cognitive impairment (MCI), 220 AD) with FBP scans (4 × 5min frames carried out 50–70 min post-injection) processed with the previous (FS v5.3) and updated (FS v7.1) ADNI pipeline to validate the previous 1.11 threshold.

We also used 295 non-overlapping ADNI-3 participants (166 cognitively normal, 96 MCI, 33 AD) with FBB scans (4 × 5min frames, 90–110 min) and contemporaneous structural MRIs processed with the updated ADNI pipeline to validate the threshold identified by the young healthy controls (FBB threshold derivation cohort).

### Centiloid validation and verification procedures

To validate our local CL pipeline implementation, we downloaded the reference PiB PET (50–70 min) dataset from GAAIN, on which the CL method was developed and replicated the level-1 CL analysis described in Klunk et al. [5]. Briefly, this dataset includes 34 young controls (YC) and 45 older adults with clinically diagnosed AD, which serve as the CL scale’s anchor points of 0 and 100 units, respectively. Of exception to the original level-1 analysis, we used a newer version of SPM (version 12; https://www.fil.ion.ucl.ac.uk/spm/software/spm12/). Local implementation of the ADNI FS v7.1 pipeline also required validation. To accomplish this, the University of Pittsburgh (UP) processed 100 FBP PET images previously analyzed by the University of California, Berkeley (UCB), using the ADNI FS v7.1 pipeline. UP- and UCB-derived summary cortical SUVR outcomes were subsequently compared.

### Data processing

#### ADNI pipeline

We recently updated the ADNI Aβ PET processing pipeline to include two major changes: (1) cortical summary and reference regions are defined using FS v7.1 and (2) mean uptake is calculated across the entire cortical summary regions (a mask made up of FS-defined frontal, cingulate, lateral parietal, and lateral temporal regions) rather than an unweighted average of the same frontal, cingulate, parietal, and lateral temporal regions; for more detail, see UCB AV45 and FBB methods summaries on the LONI Image Data Archive Website [21].

Using FBB and FBP datasets from GAAIN, we analyzed corresponding MRIs with the updated ADNI pipeline, which employs FS v7.1 to generate a native-space FS atlas for each MRI [22] (https://surfer.nmr.mgh.harvard.edu/fswiki/rel7downloads). Also from GAAIN, we downloaded FBB PET scans as static images acquired 90–110 min post-injection and FBP PET scans as two 5-min frames spanning 50–60 min post-injection. We motion-corrected and averaged FBP images using PMOD (www.pmod.com). We subsequently co-registered both FBB and FBP PET images to their respective native-space MRIs in SPM12. We then sampled the PET images to assess the mean tracer uptake in reference and target cortical regions, normalized to the whole cerebellum. We also created SUVRs for each FBB and FBP PET scan using a composite reference region, calculated as an unweighted average of FS-defined whole cerebellum, brainstem, and eroded subcortical white matter as previously described for use in longitudinal analyses [12].

We additionally calculated global FBB SUVRs for the threshold derivation sample images (images acquired from LMI) using a co-registered MRI and the same updated FS v7.1-based PET pipeline and SUVR calculation approach described above.

#### Centiloid pipeline

We downloaded PiB images from GAAIN that were concurrent with FBB and FBP data and analyzed using the standard CL pipeline, as described by Klunk et al. [5].

#### ADNI-Centiloid conversion

Using the GAAIN datasets, we correlated the corresponding standard CL pipeline-derived PiB SUVRs to ADNI FS v7.1 pipeline-derived FBB and FBP SUVRs, yielding linear equations. From these equations, we then used the intercept (^Tracer^*b*_NS_) and slope (^Tracer^*m*_NS_) to convert individual FBB and FBP SUVRs (^Tracer^SUVR_IND_) to equivalent “PiB calculated” SUVRs (^PiB-Calc^SUVR_IND_), as described by Klunk et al. [5] Section 2.2.3.1:

1 $$ {}^{\mathrm{PiB}-\mathrm{Calc}}{\mathrm{SUVR}}_{\mathrm{IND}}=\left({{}^{\mathrm{Tracer}}\mathrm{SUVR}}_{\mathrm{IND}}-{{}^{\mathrm{Tracer}}b}_{\mathrm{NS}}\right)/{{}^{\mathrm{Tracer}}m}_{\mathrm{NS}} $$

We subsequently converted the “calculated” PiB SUVRs to CL units (^PiB-Calc^CL) using the anchor points derived from the GAAIN 34 YC-0 (1.01) and 45 AD-100 (2.08) PiB SUVRs as described in Klunk et al. [5] Section 2.2.3.1:

2 $$ {}^{\mathrm{PiB}-\mathrm{Calc}}\mathrm{CL}=\frac{100\left({}^{\mathrm{PiB}-\mathrm{Calc}}{\mathrm{SUVR}}_{\mathrm{IND}}-1.01\ \right)}{\left(2.08-1.01\right)} $$

We then correlated “PiB calculated” CL to their respective FBB or FBP SUVRs and created the equations required to convert ADNI FS v7.1 pipeline-derived FBB or FBP SUVRs directly to CL units.

For each tracer, this process was performed twice: first using FBB and FBP SUVRs normalized to the whole cerebellum and then using those normalized to the composite reference region.

#### Threshold derivation and validation

We calculated FBB positivity thresholds using two approaches. We used the LMI-derived FBB threshold derivation cohort to determine the upper limit (mean + 2SD) of cortical Aβ accumulation relative to the whole cerebellum in a young, cognitively normal sample. We then used the ADNI FBB threshold validation cohort to carry out a data-driven approach (Gaussian mixture model (GMM); R package mixtools). We used whole cerebellum-normalized cortical FBB SUVRs to model 1000 GMM iterations across each of 1–4 mixture components and calculated the median Akaike Information Criterion (AIC) for each number of mixture components in order to select the optimal GMM. The positivity threshold was defined by the mean + 2SD of the lower distribution. Finally, we used 1292 ADNI participants with baseline FBP scans to compare FS v5.3 (previous) and FS v7.1 (updated) cortical FBP SUVRs and validate our previously established 1.11 threshold.

## Results

### Validation

**Fig. 1:**
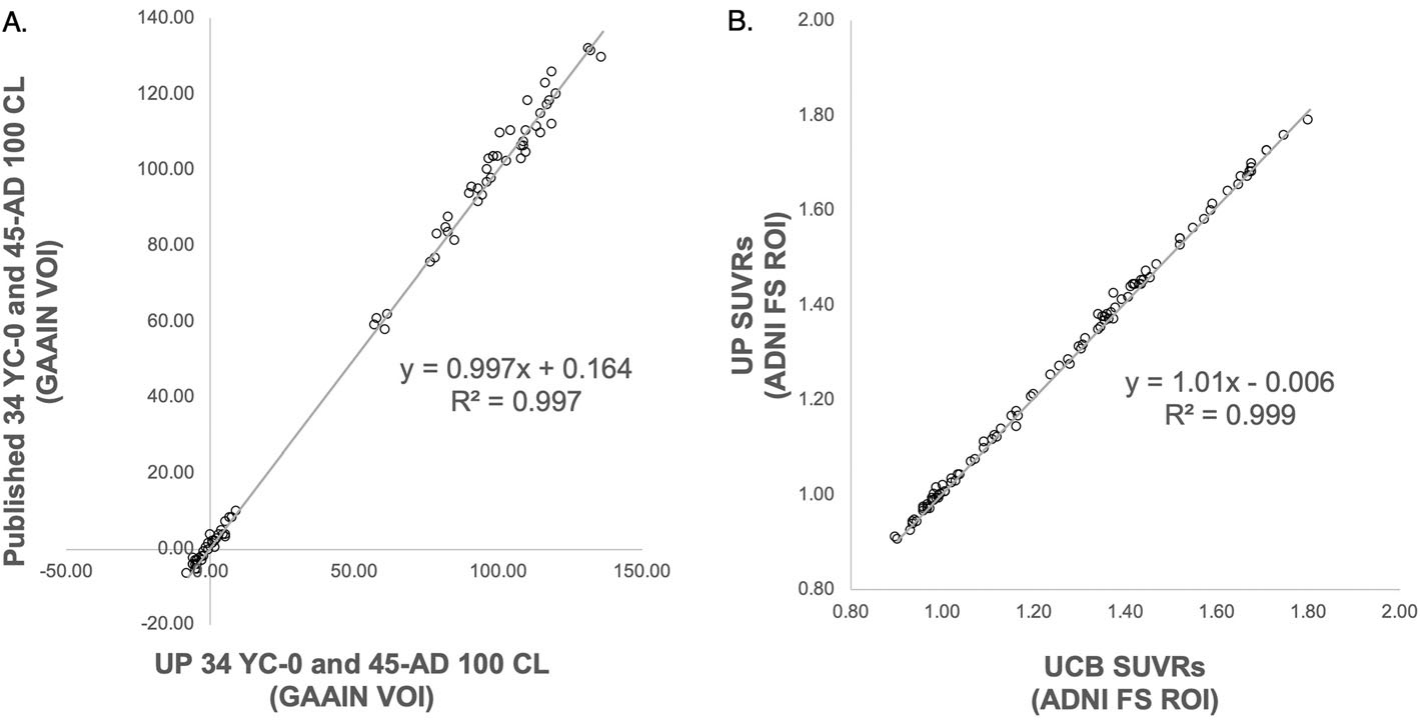
**a** CL outcomes derived from UP’s level-1 analysis of the GAAIN 34 YC-0 and 45 AD-100 scans vs. published CL values. The equation and *R*^2^ indicate that the standard CL pipeline was appropriately replicated. **b** FBP SUVRs derived from UP’s implementation of the ADNI FS v7.1 pipeline vs. SUVRs provided by UCB; FBP scans were provided to UP by UCB. The equation and *R*^2^ indicate appropriate local implementation of the ADNI FS v7.1 pipeline

Linear regression of local standard CL level-1 outcomes against published CL outcomes yielded a fit equation with a slope and correlation coefficient near unity (*y* = 0.997*x* + 0.164, *R*^2^ > 0.997; Fig. 1a), thus exceeding minimum acceptance criteria for local implementation [5]. This high concordance additionally verified that results derived from SPM12 did not differ from those published by Klunk et al. [5]. We also validated local implementation of the ADNI FS v7.1 pipeline using the UCB dataset by performing a linear regression of SUVR outcomes, yielding a high degree of agreement (*y* = 1.01*x* + 0.006, *R*^2^ > 0.999; Fig. 1b).

### Conversion equations

**Fig. 2:**
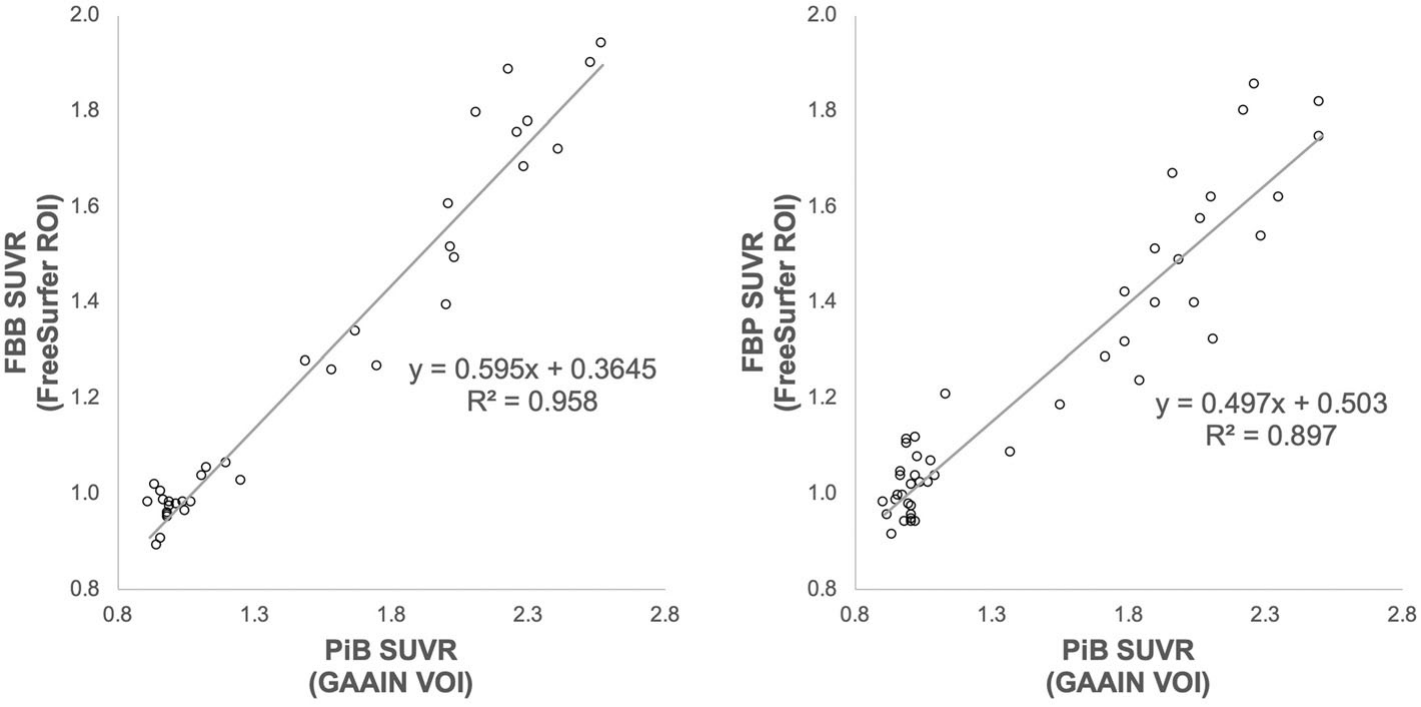
Linear regressions of FBB (left) and FBP (right) whole cerebellum-normalized SUVRs derived from ADNI FS v7.1 pipeline against PiB SUVRs derived from the standard CL pipeline. A linear conversion from these regressions was used to create “Calculated” PiB SUVRs from FBB and FBP SUVRs

Linear regressions of ADNI FS v7.1 pipeline FBB and FBP whole cerebellum-normalized SUVRs against standard CL pipeline PiB SUVRs resulted in an acceptable level of correlation (*R*^2^ > 0.7; Fig. 2) [5]. The equations used to scale ADNI FS v7.1 pipeline-derived FBB (^FBB^SUVR_IND_) and FBP (^FBP^SUVR_IND_) whole cerebellum-normalized SUVRs to calculated PiB SUVRs (^PiB-Calc^SUVR_IND_) are as follows:

3 $$ \mathrm{FBB}:{}^{\mathrm{PiB}-\mathrm{Calc}}{\mathrm{SUVR}}_{\mathrm{IND}}=\left({{}^{\mathrm{FBB}}\mathrm{SUVR}}_{\mathrm{IND}}-0.365\right)/0.595 $$

4 $$ \mathrm{FBP}:{}^{\mathrm{PiB}-\mathrm{Calc}}{\mathrm{SUVR}}_{\mathrm{IND}}=\left({}^{\mathrm{FBP}}{\mathrm{SUVR}}_{\mathrm{IND}}-0.503\right)/0.497 $$

The direct equation for converting FBB ADNI FS v7.1 whole cerebellum-normalized SUVRs (SUVR_FBB_) to CL units is:

5 $$ \mathrm{CL}=157.15\times {\mathrm{SUVR}}_{\mathrm{FBB}}-151.87 $$

The direct equation for converting FBP ADNI FS v7.1 whole cerebellum-normalized SUVRs (SUVR_FBP_) to CL units is:

6 $$ \mathrm{CL}=188.22\times {\mathrm{SUVR}}_{\mathrm{FBP}}-189.16 $$

The correlations between ADNI FS v7.1 pipeline FBB and FBP composite-normalized SUVRs against standard CL pipeline PiB SUVRs additionally surpassed the minimum acceptable requirements (Supplemental Figure 1) [5].

The direct equation for converting FBB ADNI FS v7.1 composite-normalized SUVRs (SUVR_FBB_) to CL units is:

7 $$ \mathrm{CL}=244.20\times {\mathrm{SUVR}}_{\mathrm{FBB}}-170.80 $$

The direct equation for converting FBP ADNI FS v7.1 composite-normalized SUVRs (SUVR_FBP_) to CL units is:

8 $$ \mathrm{CL}=300.66\times {\mathrm{SUVR}}_{\mathrm{FBP}}-208.84 $$

### Comparison of ADNI FS v7.1 pipeline CL values (FBB, FBP) to standard CL pipeline values (PIB)

FBB and PiB mean and standard deviation CL values for the young adult controls were 0.25 ± 4.83 and −1.21 ± 3.57, respectively, yielding a standard deviation ratio (SD_FBB_/SD_PiB_) of 1.35. The FBP mean and standard deviation of CL values for the young adult controls were −0.43 ± 9.99 while those of PiB were −1.11 ± 3.45. This yielded a standard deviation ratio (SD_FBP_/SD_PiB_) of 2.89.

Detailed summary statistics for the comparison of FBB, FBP, and PiB SUVR and CL values are shown in Table 1.

**Table 1:** SUVR and CL values for both FBB and FBP and their respective PiB scans. FBB and FBP scans were processed using the ADNI FS v7.1 pipeline and PiB scans were processed using the standard CL pipeline

[^**18**^F]florbetaben cohort				[^**18**^F]florbetapir cohort			
**[**^**11**^**C]PiB**				**[**^**11**^**C]PiB**			
		SUVR	CL			SUVR	CL
Elderly	Mean	1.72	66.00	Elderly	Mean	1.62	57.26
	SD	0.57	52.91		SD	0.55	51.07
	CV (%)	33			CV (%)	34	
YC	Mean	1.00	−1.21	YC	Mean	1.00	−1.11
	SD	0.04	3.57		SD	0.04	3.45
	CV (%)	4			CV (%)	4	
**[**^**18**^**F]florbetaben**				**[**^**18**^**F]florbetapir**			
		SUVR	CL			SUVR	CL
Elderly	Mean	1.38	65.40	Elderly	Mean	1.31	57.01
	SD	0.35	55.29		SD	0.29	55.00
	CV (%)	25			CV (%)	22	
YC	Mean	0.97	0.25	YC	Mean	1.00	−0.43
	SD	0.03	4.83		SD	0.05	9.99
	CV (%)	3			CV (%)	5	
FBB/PiB SD ratio 1.35				FBP/PiB SD ratio 2.89			

Summary statistics comparing FBB, FBP, and PiB CL values where FBB and FBP SUVRs are normalized to the composite reference region are outlined in Supplemental Table 2.

### FBB and FBP positivity thresholds

**Fig. 3:**
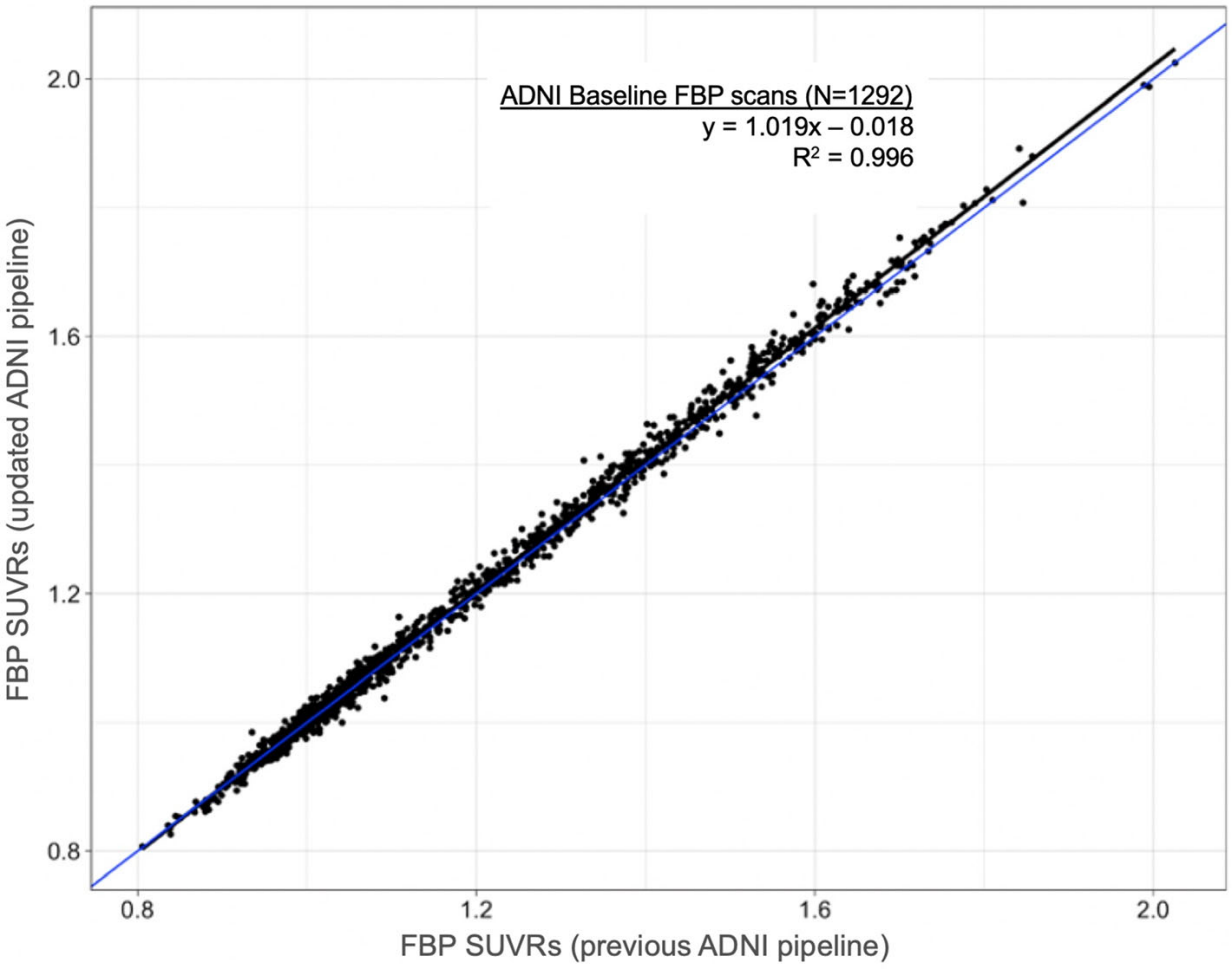
All baseline ADNI FBP scans (*N*=1292) were analyzed with both the previous and updated ANDI pipelines. The best-fit linear regression line (black) was used to confirm that the previously validated FBP threshold (cortical summary/whole cerebellum SUVR=1.11) is unchanged with the FS v7.1 pipeline

As our previously validated FBP threshold (1.11) was based on a processing pipeline that incorporated FS v5.3, we examined cortical FBP SUVRs of 1292 baseline ADNI FBP scans analyzed using both FS v5.3 and FS v7.1 to characterize any effects related to FS version or the new weighting as described in the section “ADNI pipeline” section above (Fig. 3; *y*= 1.019*x* − 0.018, *R*^2^=0.996). This resulted in no change to the 1.11 threshold (20 CL).

The mean + 2SD of whole cerebellum-normalized cortical FBB SUVRs in the LMI young controls (mean=1.012, SD=0.033) resulted in a threshold of 1.08 (18 CL).

**Fig. 4:**
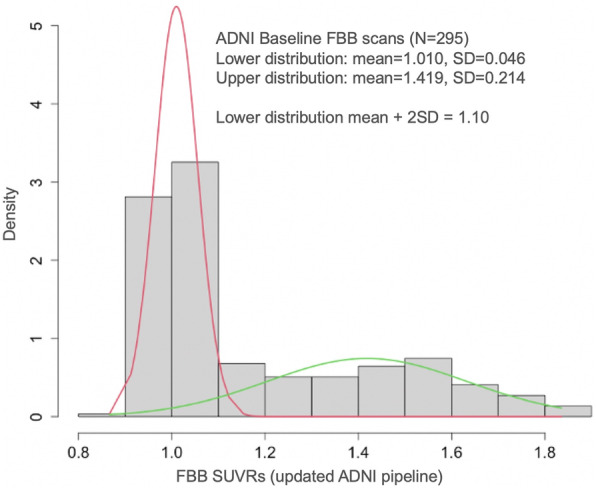
Gaussian mixture modeling distributions for ADNI baseline FBB scans analyzed with the updated ADNI pipeline

We also used a data-driven approach (GMM) to model all available whole cerebellum-normalized FBB SUVRs in ADNI. Model selection resulted in a GMM with 2 mixture components (Fig. 4; lower distribution mean=1.010 + 0.046) with a lower distribution mean + 2SD value of 1.10 (21 CL). A comparison of a single mixture component did not improve model fit according to AIC, and the use of 3–4 mixture components resulted in comparable AIC and positivity threshold as the 2-component model.

The final GMM-derived threshold (1.10, 21 CL) was similar to the threshold based on young controls (1.08; 18 CL). We chose 1.08 because it is based on an ADNI-independent sample and because the FBP threshold was defined by a similar young control sample, making the threshold derivation methods for the two ligands congruent with one another.

## Discussion

Variability across radiotracers, scanners, and analysis pipelines limits the comparison of Aβ PET measurements across studies. To address these issues and facilitate comparison of Aβ burden across different ligands, we used a variety of datasets to derive CL transformations for ADNI FS v7.1 FBB and FBP SUVRs. We applied these CL transformations to validate FBB and FBP thresholds that were derived using congruent approaches and that can be used to determine Aβ positivity in datasets that include both tracers. While the primary objectives of this work were to calculate the CL conversions and thresholds for ADNI FS v7.1-derived SUVRs normalized to the whole cerebellum (as is done for cross-sectional studies), we additionally defined CL conversions for SUVRs normalized to a composite reference region, which is recommended for longitudinal studies using FBP. All of these findings are immediately applicable to the thousands of current and anticipated SUVRs acquired using methods compatible with ADNI. More broadly, our CL transformations can be applied to any FBB and FBP PET image data that were (1) collected according to ADNI-like acquisition and pre-processing protocols and (2) analyzed using the updated PET pipeline described here, which is based on a native-space, FS-based quantification approach.

The FBP and FBB thresholds were based on the upper limit of cortical uptake in whole cerebellum-normalized SUVRs in young control samples. For FBP, we confirmed that our previously validated threshold (1.11, 20 CL) that was transformed from Avid-acquired young control data [3, 19] was unchanged using the updated ADNI pipeline. For FBB, we analyzed young control data acquired by LMI [20] with the updated ADNI pipeline to derive a threshold (1.08, 18 CL for SUVRs normalized to the whole cerebellum) and verified a similar result using a data-driven approach with the existing ADNI FBB sample (1.10, 21 CL for SUVRs normalized to the whole cerebellum). While these values are similar, the 1.08 (18 CL) threshold is preferred as it was derived in a sample independent from ADNI and because it is based on healthy individuals who are free of Aβ burden, making it methodologically congruent with the FBP threshold. Previous studies have developed FBB thresholds using histopathological confirmation of Aβ in postmortem brain tissue and other methods that have primarily focused on the detection of clinical characteristics and/or Aβ [16, 17, 23]. These thresholds may be less sensitive to early elevations in Aβ burden compared to the approach used here, which relies on cortical Aβ and associated Aβ variability in healthy controls with no Aβ burden. In addition, many previous FBB studies have used the cerebellar cortex as a reference region, resulting in lower reference region estimates and a higher cortical Aβ SUVR threshold, as opposed to the whole cerebellum (white and gray matter), which results in higher reference region estimates and a lower cortical SUVR as reported here.

Previous studies have demonstrated that both FBB and FBP SUVRs are appropriate for conversion to CL units using the standard CL analysis pipeline [5, 10, 11]. The analysis presented in this work confirms these findings. In both FBB and FBP cohorts, we observed a strong correlation between ADNI FS v7.1 pipeline-derived whole cerebellum-normalized SUVRs and their respective standard CL pipeline-derived PiB SUVRs (*R*^2^=0.958 for FBB; *R*^2^=0.897 for FBP).

The equation describing the linear regression of ADNI FS v7.1 FBB SUVRs normalized to the whole cerebellum and the standard CL pipeline PiB SUVRs (Eq. 3) indicates that FBB has a narrower dynamic range compared to PiB (slope<1), which is consistent with the previous literature [24]. The increased variance we observed in the FBB CL units relative to the PiB CL units is likely to be due to differences between tracers. Rowe et al. [10] similarly found increased variance in FBB CL units compared to PiB CL units when SUVRs for both tracers were derived from the standard CL pipeline. However, compared to our findings, this group reported less precision in FBB CL units (SD=6.81) and a greater standard deviation ratio (1.96), suggesting that compared to the CL pipeline, the ADNI pipeline introduces less variance.

FBP has previously been reported to have about one-half of the dynamic range of PiB [3]. The slope of the equation describing the linear regression of ADNI FS v7.1 FBP SUVRs normalized to the whole cerebellum and CL pipeline PiB SUVRs (Eq. 4) reflects this. We also found more variability in FBP CL values in young controls compared to PiB CL values. Navitsky et al. [11] also found greater variance in CL units converted from standard CL pipeline-derived FBP SUVRs compared to those from PiB. This study also reported larger SD (12.07) and standard deviation ratio (3.96) for YC CL units when compared to the present work, so the increased variance that we observed is likely due to differences between FBP and PiB as the ADNI pipeline appears to introduce less variance than the standard CL pipeline. These findings together reinforce the fact that conversion to CL values does not improve the precision of [^18^F] tracers relative to PiB.

The equations that describe whole cerebellum-normalized SUVR-to-CL units for both FBB and FBP (Eqs. 5 and 6) are markedly different from those previously published for the same tracers [10, 11]. Importantly, such work has transformed SUVRs derived from the standard CL pipeline to CL units whereas we transformed SUVRs derived from FS v7.1. Sizes of the cortical regions between the two pipelines differ (Supplemental Table 2), as does sampled tissue (Supplemental Figure 2). The equations describing conversion from composite region-normalized SUVR-to-CL units (Eqs. 7 and 8) are even more different from previously published transformations, likely due to both the different cortical and reference regions. While the use of a reference region containing subcortical white matter reduces the dynamic range of amyloid PET SUVRs, several studies have demonstrated that it optimizes longitudinal PiB and FBP reliability [12–15]. It is important to note that the transformation equations described in this manuscript are not appropriate for converting any other type of SUVR to CL units; they only are only suitable for FBB and FBP outcomes derived from the ADNI FS v7.1 pipeline using either the whole cerebellum or composite as reference region.

It is worth noting that in the GAAIN datasets, relative standard deviations of FBP-to-PiB CL units are more than two times greater than that of FBB-to-PiB CL units. The age distribution is comparable between the two cohorts [10, 11], and the mean and SD of PiB SUVR and CL units are almost identical between young controls. However, the standard deviation ratio of whole cerebellum-normalized SUVRs in the FBP cohort is 67% greater than that of the FBB cohort. Thus, the disparity in standard deviation ratios between FBB and FBP CL units may be attributed to differences in dynamic ranges relative to PiB. Alternatively, the increased standard deviation ratio of FBP may be due to scanner differences, as FBP CL data was collected on three different PET scanners at three different sites [11], whereas FBB CL data was collected on two PET scanners at a single site [10].

Our thresholds expressed in CL units are consistent with recent reports for FBB [18, 25] and FBP [26] specifically, and for Aβ burden studies in general [25–27]. Such studies have described CL thresholds that range from 12 to 35 depending on the stringency of the threshold standard, quantification approach, and tracer; lower thresholds emphasize early detection and higher thresholds maximize specificity [18]. Using positive visual reads as the standard, our CL thresholds (FBB 18 CL; FBP 20 CL) fall slightly lower than the published range of 25–35 [25]. However, using CSF [26] and the presence of plaques with histopathological examination as the standard, our thresholds are on the high end of the reported ranges. That is, in one neuropathological validation study, Rowe et al. [25] reported a range of 15–20 CL with FBB or PiB. Additionally, La Joie et al. [27] reported a threshold of 12 CL using PiB for detection of moderate to frequent plaques and 24 CL for identification of AD neuropathologic change (a composite score). It should also be noted that in another study that used an MRI-based quantification approach similar to ours, Dore et al. [18] reported a range of 22–28 FBB-derived CL and thus, our range is comparatively lower.

A strength of the present study is its multi-scanner nature, since ADNI pre-processing allows data from multiple sites to be merged despite differences in scanners, reconstruction methods, and spatial resolution.

## Limitations

Between-scanner differences were not taken into account in the GAAIN cohorts, which likely contributed to noise in the CL transformations. However, harmonization methods were not included in the original CL methods or in any subsequent work describing CL calculations and, thus, are beyond the scope of this paper. Still, the ADNI and LMI data were harmonized, internally consistent, and yielded CL values similar to that of previous literature, leading us to believe that the between-scanner differences in the GAAIN data are not of major concern.

## Conclusions

The use of different Aβ PET radiotracers leads to quantitative Aβ burden estimates that are comparable, but the data are not interchangeable. To facilitate the standardization of Aβ burden estimates, we determined transformation equations necessary to convert whole cerebellum-normalized (cross-sectional) and composite-normalized (longitudinal) SUVRs measured with FBB or FBP to CL units. We also established corresponding Aβ positivity thresholds that are numerically similar to one another and also congruent in that they were derived from young control samples that are independent of the ADNI dataset. These conversion equations and corresponding thresholds are applicable to FBB or FBP SUVRs acquired and processed using procedures consistent with ADNI. These procedures have been adopted by other ongoing multisite studies [7, 8], suggesting that the thresholds and transformations reported here can be broadly applied. Our work ultimately promotes harmonization of Aβ PET outcomes expressed in CL units but acquired and analyzed using different radiotracers, performance sites, imaging equipment, and analysis pipelines.

## Supplementary Information

**Additional file 1: Supplemental Table 1.** SUVR and CL values for both FBB and FBP and their respective PiB scans. FBB and FBP scans were processed using the ADNI FS v7.1 pipeline and normalized to the composite reference region: an unweighted average of whole cerebellum, pons, and eroded subcortical white matter. PiB scans were processed using the standard CL pipeline. **Supplemental Table 2.** VOI volumes for each set of scans used for creating the CL transformation equations. **Supplemental Figure 1.** Linear regressions of FBB (left) and FBP (right) SUVRs derived from ADNI FS v7.1 pipeline using the composite reference region against PiB SUVRs derived from the standard CL pipeline. A linear conversion from these regressions was used to create “Calculated” PiB SUVRs from FBB and FBP SUVRs. **Supplemental Figure 2. A.** CL cortex VOI overtop MRI and corresponding PiB PET; **B.** ADNI FS v7.1 cortex ROI overtop MRI and corresponding FBB PET.

## Data Availability

Datasets analyzed during this study were acquired from the ADNI database (adni.loni.ucla.edu) and the GAAIN database (www.gain.org/centiloid-project).

## Abbreviations

Aβ: β-Amyloid
AD: Alzheimer’s disease
ADNI: Alzheimer’s Disease Neuroimaging Initiative
CL: Centiloid
CT: Computed tomography
FBB: [^18^F]florbetaben
FBP: [^18^F]florbetapir
FS: FreeSurfer
GAAIN: Global Alzheimer’s Association Interactive Network
LMI: Life Molecular Imaging
MRI: Magnetic resonance image
PET: Positron emission tomography
PiB: [^11^]C-Pittsburgh Compound-B
RAMLA: Row-action maximum likelihood algorithm
SPM: Statistical Parametric Mapping
SUVR: Standardized uptake value ratio
UCB: University of California Berkeley
UP: University of Pittsburgh
YC: Young control
