# Supplementary material for: Validation of amyloid PET positivity thresholds in centiloids: a multisite PET study approach
Source: Alzheimers Res Ther. 2021 May 10;13:99. doi: 10.1186/s13195-021-00836-1 (PMC8111744; doi:10.1186/s13195-021-00836-1)

**SUPPLEMENTARY INFORMATION**

**SUPPLEMENTAL TABLE 1.** SUVR and CL values for both FBB and FBP and their respective PiB scans. FBB and FBP scans were processed using the ADNI FS v7.1 pipeline and normalized to the composite reference region: an unweighted average of whole cerebellum, pons, and eroded subcortical white matter. PiB scans were processed using the standard CL pipeline.

| **[^18^F]florbetaben Cohort** | | | | **[^18^F]florbetapir Cohort** | | | |
| --- | --- | --- | --- | --- | --- | --- | --- |
| **[^11^C]PiB** | | | | **[^11^C]PiB** | | | |
|  |  | SUVR | CL |  |  | SUVR | CL |
| Elderly |  |  |  | Elderly |  |  |  |
|  | mean | 1.72 | 70.00 |  | mean | 1.62 | 57.26 |
|  | SD | 0.57 | 52.91 |  | SD | 0.55 | 51.07 |
|  | CV (%) | 33 |  |  | CV (%) | 34 |  |
| YC |  |  |  | YC |  |  |  |
|  | mean | 1.00 | -1.22 |  | mean | 1.00 | -1.11 |
|  | SD | 0.04 | 3.57 |  | SD | 0.04 | 3.45 |
|  | CV (%) | 4 |  |  | CV (%) | 4 |  |

| **[^18^F]florbetaben:**  ***Composite reference*** | | | | **[^18^F]florbetapir:**  ***Composite reference*** | | | |
| --- | --- | --- | --- | --- | --- | --- | --- |
|  |  | SUVR | CL |  |  | SUVR | CL |
| Elderly |  |  |  | Elderly |  |  |  |
|  | mean | 0.97 | 66.48 |  | mean | 0.88 | 56.29 |
|  | SD | 0.22 | 53.52 |  | SD | 0.19 | 56.26 |
|  | CV (%) | 23 |  |  | CV (%) | 21 |  |
| YC |  |  |  | YC |  |  |  |
|  | mean | 0.69 | -2.42 |  | mean | 0.70 | 1.89 |
|  | SD | 0.02 | 5.33 |  | SD | 0.04 | 12.41 |
|  | CV (%) | 3 |  |  | CV (%) | 6 |  |

| FBB/PiB SD Ratio (*composite*): 1.49 | FBP/PiB SD Ratio (*composite*): 3.59 |
| --- | --- |

**SUPPLEMENTAL TABLE 2.** VOI volumes for each set of scans used for creating the CL transformation equations.

|  | Mean cortex VOI volume (cm^3^) | Range of VOI volumes (cm^3^) |
| --- | --- | --- |
| PiB (CL) | 234.62 | -- |
| FBB (FS v7.1) | 272.58 | 217.33, 333.90 |
| FBP (FS v7.1) | 273.15 | 175.21, 346.23 |

**SUPPLEMENTAL FIGURE 1.** Linear regressions of (FBB (left) and FBP (right) SUVRs derived from ADNI FS v7.1 pipeline using the composite reference region against PiB SUVRs derived from CL pipeline. A linear conversion from these regressions was used to create “Calculated” PiB SUVRs from FBB and FBP SUVRs.


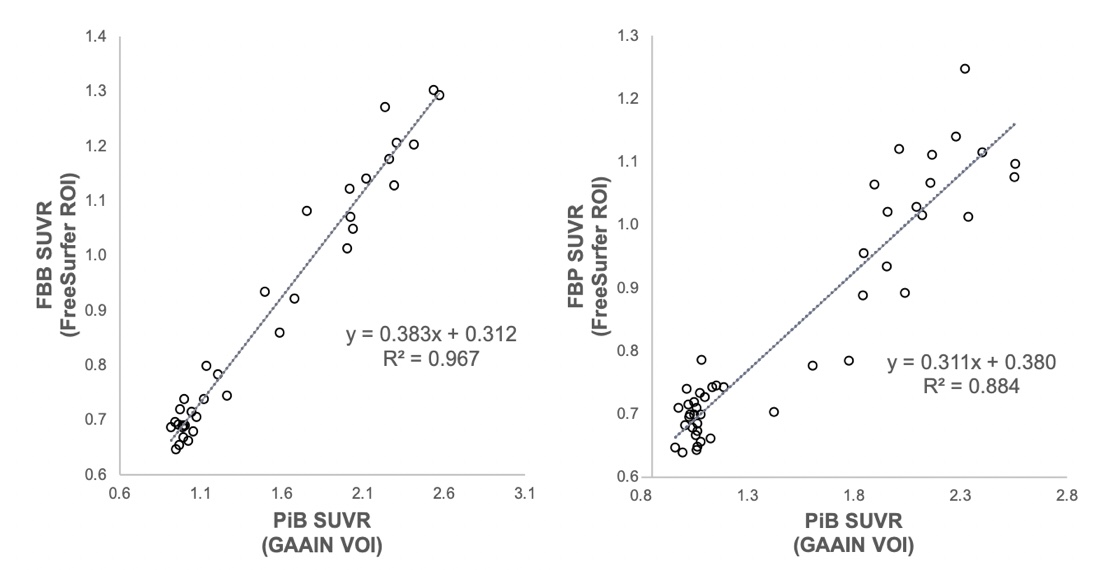


**SUPPLEMENTAL FIGURE 2. A.** CL cortex VOI overtop MRI and corresponding PiB PET; **B.** ADNI FS v7.1 cortex ROI overtop MRI and corresponding FBB PET.


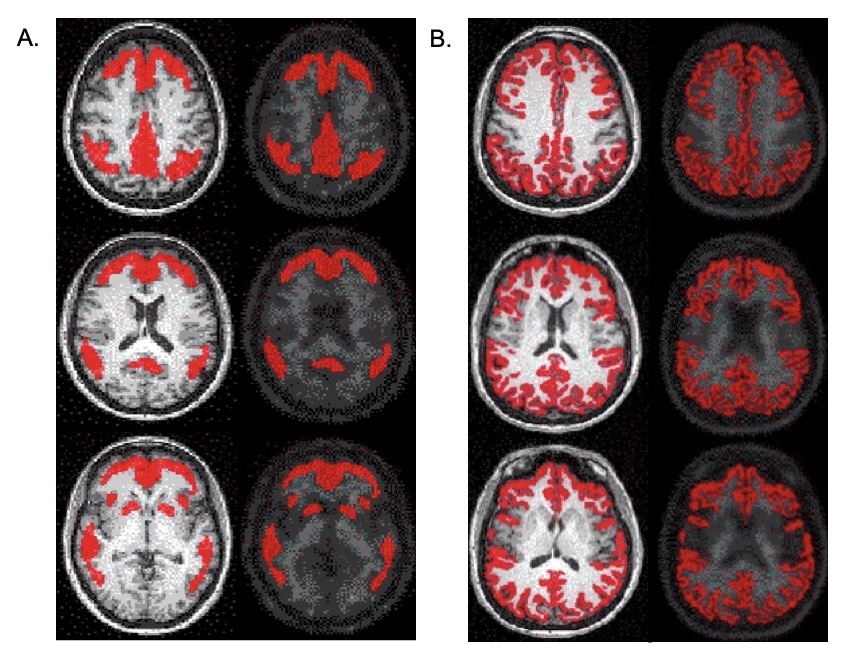

Supplement: Supplementary file 1 — Additional file 1: Supplemental Table 1. SUVR and CL values for both FBB and FBP and their respective PiB scans. FBB and FBP scans were processed using the ADNI FS v7.1 pipeline and normalized to the composite reference region: an unweighted average of whole cerebellum, pons, and eroded subcortical white matter. PiB scans were processed using the standard CL pipeline. Supplemental Table 2. VOI volumes for each set of scans used for creating the CL transformation equations. Supplemental Figure 1. Linear regressions of FBB (left) and FBP (right) SUVRs derived from ADNI FS v7.1 pipeline using the composite reference region against PiB SUVRs derived from the standard CL pipeline. A linear conversion from these regressions was used to create “Calculated” PiB SUVRs from FBB and FBP SUVRs. Supplemental Figure 2. A. CL cortex VOI overtop MRI and corresponding PiB PET; B. ADNI FS v7.1 cortex ROI overtop MRI and corresponding FBB PET. [file 13195_2021_836_MOESM1_ESM.docx]
